# Application of statistical process control to qualitative molecular diagnostic assays

**DOI:** 10.3389/fmolb.2014.00018

**Published:** 2014-11-05

**Authors:** Cathal P. O'Brien, Stephen P. Finn

**Affiliations:** ^1^Cancer Molecular Diagnostics, Labmed Directorate, St. James's HospitalDublin, Ireland; ^2^Department of Histopathology, St. James's HospitalDublin, Ireland; ^3^Department of Histopathology, Trinity College DublinIreland

**Keywords:** quality improvement, molecular diagnostics, statistical process control, laboratory improvement, biomarkers

## Abstract

Modern pathology laboratories and in particular high throughput laboratories such as clinical chemistry have developed a reliable system for statistical process control (SPC). Such a system is absent from the majority of molecular laboratories and where present is confined to quantitative assays. As the inability to apply SPC to an assay is an obvious disadvantage this study aimed to solve this problem by using a frequency estimate coupled with a confidence interval calculation to detect deviations from an expected mutation frequency. The results of this study demonstrate the strengths and weaknesses of this approach and highlight minimum sample number requirements. Notably, assays with low mutation frequencies and detection of small deviations from an expected value require greater sample numbers to mitigate a protracted time to detection. Modeled laboratory data was also used to highlight how this approach might be applied in a routine molecular laboratory. This article is the first to describe the application of SPC to qualitative laboratory data.

## Introduction

Within the field of molecular pathology and molecular diagnostics, qualitative assays often suffer from a limited repertoire of methods for monitoring process performance over time. This is quite the opposite to high-throughput departments such as clinical biochemistry which run controls throughout the day and plot the performance of these controls on Levey-Jennings charts or an equivalent form of monitoring chart in a process termed statistical process control (SPC) (Levey and Jennings, 1950). The characteristics of clinical biochemistry that render it suitable for SPC are twofold; firstly, the laboratories have the throughput to generate statistically relevant data and can add run controls to routine work; secondly, clinical biochemistry tends to generate quantitative data which lends itself to plotting on control charts.

With the emergence of targeted therapies in hematological malignancy and solid tumors, many molecular assays are now processed in numbers sufficient to permit some form of SPC and this has been achieved for quantitative molecular assays (Liang et al., 2008). However, the literature regarding SPC or an equivalent technique for qualitative molecular diagnostic assays is scant and those wishing to apply statistically valid monitoring to qualitative assays have a limited range of techniques to choose from.

While some articles in the field of quality control do make reference to qualitative observations (Spanos and Chen, 1997) these approaches may be difficult to translate directly to the clinical laboratory. A relatively simple approach that may be applied to any laboratory with sufficient throughput is the monitoring of mutation frequencies in tested samples. By combining point estimates of mutation frequency with statistically informative confidence intervals the laboratory can monitor process variations and performance relative to a known reference point over time.

In this article we assess the applicability of SPC to qualitative molecular pathology assays. It is our contention that a confidence interval of the observed mutation frequency may be compared to an expected value to give a reliable indicator of process performance. If a laboratory finds the mutation frequency confidence interval to lie outside of an expected value or range of values, this would trigger further investigative action and troubleshooting and may permit earlier detection of assay or protocol deviations that may be detrimental to patient care. By using statistical models we highlight the strengths and weaknesses of this approach and use modeled data to demonstrate how this may be applied in a routine laboratory. Significantly, we are also able to calculate minimum sample numbers necessary to apply this technique in a given clinical scenario with clear implications for laboratories seeking to implement high standards of process control.

## Materials and methods

### Modeling functional properties of statistical process control

The approach taken for this analysis used a point estimate of mutation frequency and a confidence interval to guide the interpretation of the point frequency estimate relative to a prior frequency estimate. Based on this approach, should the confidence limits of the point frequency estimate fail to overlap the prior frequency estimate, this would act as a trigger for further investigative action. A confidence interval of 95% was chosen and calculated using the Clopper-Pearson method (Clopper and Pearson, 1934).

The Clopper–Pearson confidence interval estimate is used to calculate binomial confidence intervals using the cumulative probabilities of the binomial distribution. The calculation is written in Equation (1) below.

(1) $$\left\{\emptyset |P[Bin(n;\emptyset )\leq X|>\frac{\emptyset }{2}\right\}\cap \left\{\emptyset |P[Bin\left(n;\emptyset \right)\geq X|>\frac{\emptyset }{2}\right\}$$

Where; *X* is the number of successes observed in the sample,

*Bin*(*n*; ∅ is a binomial random variable with n trials and probability of success ∅

All statistical calculations were performed using Matlab version 2014a (The Mathworks Inc, Natick, MA). The “sampsizepwr” function from the Statistics toolbox was used for sample size estimations using the binomial distribution. Sample numbers from 10 to 2000 per annum were used for model building with a statistical power of 0.8 and a default confidence interval of 0.95. Matlab scripts used for all calculations have been made available in the supplementary data file.

**Figure 1:**
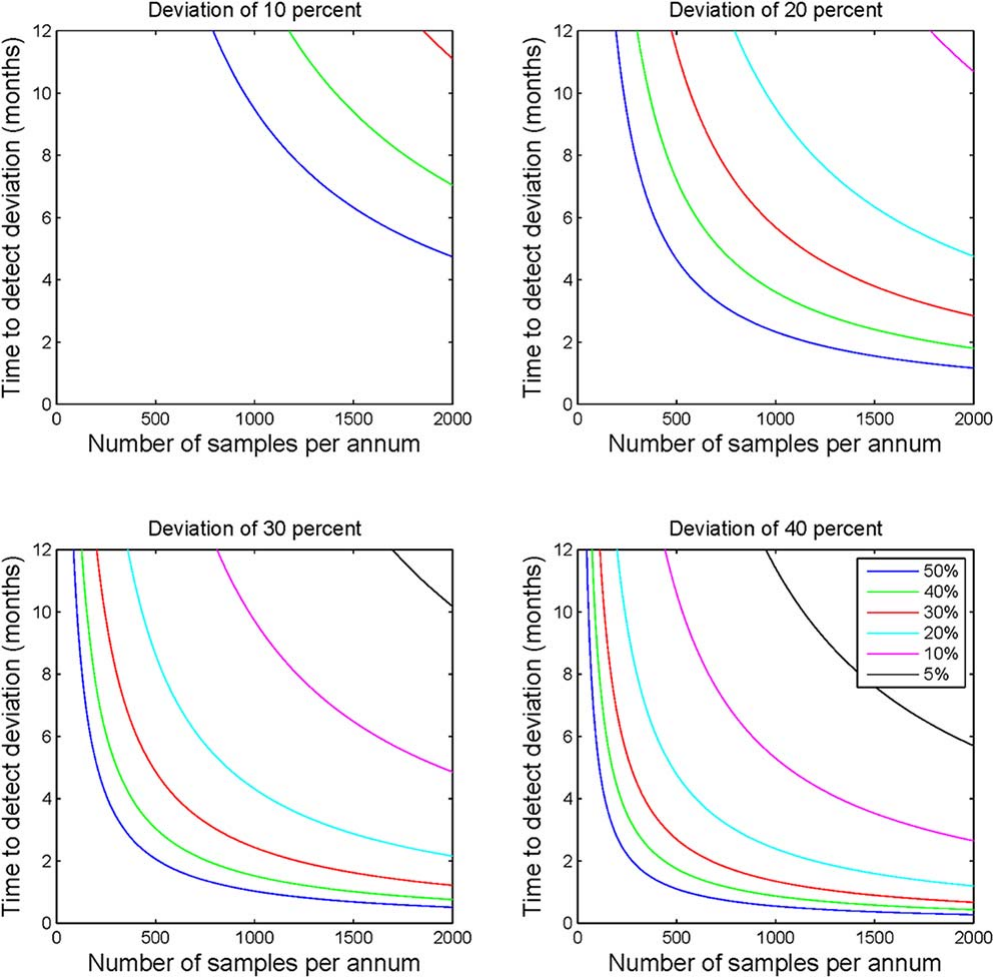
**Statistical process control sample number requirements**. Each of the four subplots demonstrate the sample numbers required to detect deviations of 10, 20, 30, and 40% for a range of prior frequency estimates ranging from 5 to 50%. This figure illustrates the non-linear relationship between sample number and applicability of the calculations to laboratory monitoring. The figure highlights the clear requirement for greater sample numbers to detect deviations where the prior frequency estimate is lower or the required detection level is lower.

To describe how the time to detection of a deviation from a prior frequency estimate might be affected by deviation size and the value of the prior frequency estimate, statistical power calculations were performed for a range of deviation sizes (10, 20, 30, and 40%) and prior frequency estimates (5, 10, 20, 30, 40, and 50%). Calculations were performed using the parameters specified above for all combinations of the deviation size and prior frequency estimates. The relationship between deviation size and mutation frequency was plotted using a series of subplots (Figure 1), and minimum recommended sample numbers were summarized using a quick reference table (Table 1).

**Table 1:**
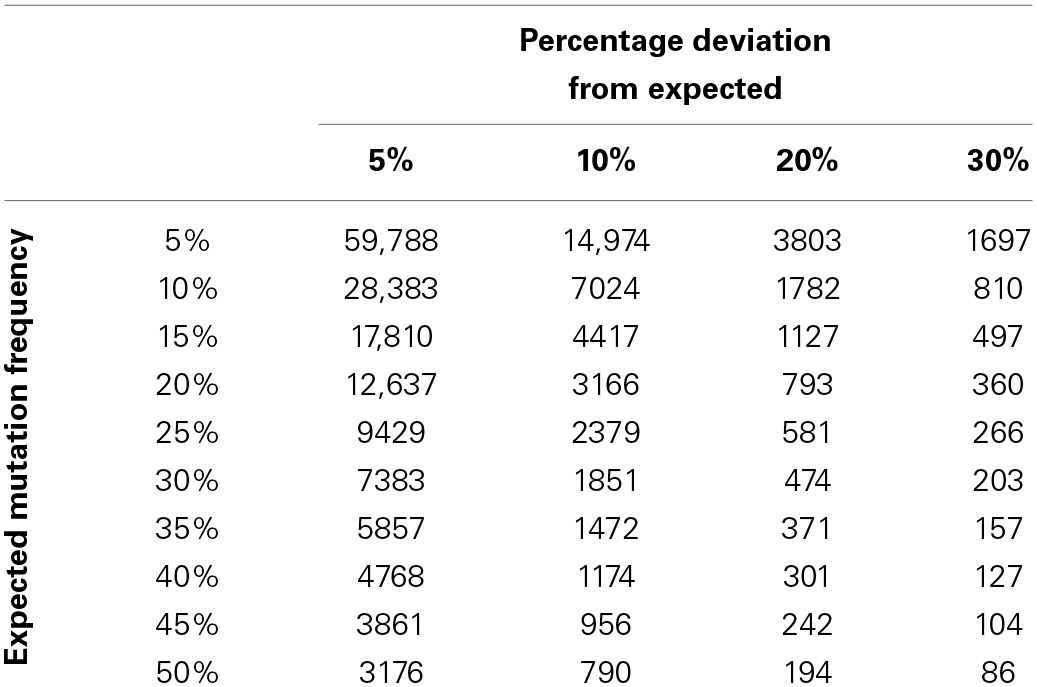
**Estimates of sample numbers required to detect deviations from an expected cut-off**.

### Modeling frequency data

A theoretical laboratory was modeled that processes 624 cases of KRAS per annum each with a prior frequency estimate of 44%. For this assay a sample requirement of 242 samples (21 weeks) to detect a deviation of 20% or more and 104 samples (9 weeks) to detect a deviation of 30% or more (Table 1). A uniform sampling interval was determined (assuming equal sample distribution throughout a year) and this was applied in a non-overlapping schema to 105 weeks of modeled data.

**Figure 2:**
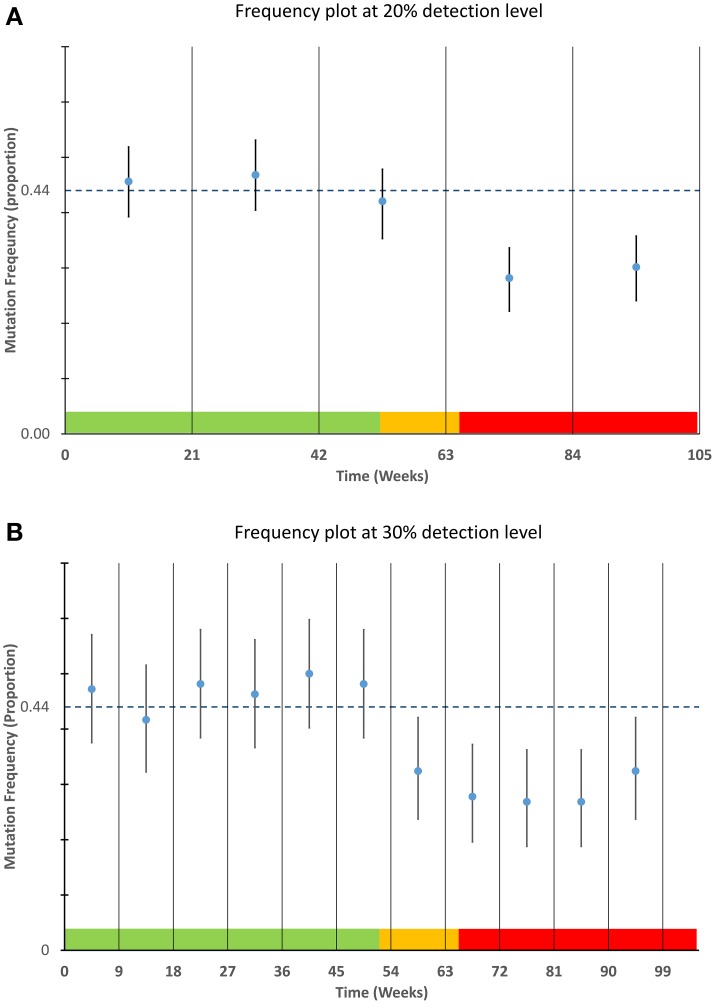
**Detection of mutation frequency deviations using frequency plots**. For each time point specified in the sampling schedule, the mean and 95% confidence interval as calculated using a Clopper-Pearson estimate was plotted along with a bar representing a prior frequency estimate. The modeled data used as described in the materials and methods is highlighted in the color bands along the lower boundary of each plot. The data within the green areas have a mean frequency of 44%, the data in the orange area are the data that are linearly decreasing and the data in the red area are stable at a lower frequency of 30%. The upper graph **(A)** is designed to detect smaller deviations of 20% but requires greater sample numbers. The lower graph **(B)** can detect deviations of approximately 30% or greater and requires fewer samples per time point.

Data were modeled to simulate a true mutation frequency of 44% for the first 52 weeks followed by a linear decline over 13 weeks to a mutation rate of 30%. To demonstrate the robustness of the process to random variation, data were modeled using a binomial distribution and random number generation routine (makedist) in Matlab®. This function models the variability inherent in a sample of a particular size (in this case 12 tests per week) is likely to vary about a given mean frequency Data from the statistical model were plotted using control charts at both 20 and 30% detection levels using Microsoft® Excel (Figure 2), for each time frame the point frequency estimate was plotted with its associated confidence interval and compared to the prior frequency estimate of 44% (or its equivalent proportion of 0.44). For ease of interpretation data from the different time stages of the model are color coded within Figure 2 with green data representing those data with a mean frequency of 44%, orange representing the decline in positivity and red representing a new stable mutation frequency of 30%.

## Results

### SPC requires adequate throughput

Analysis of the modeled data for sample throughput, proportional deviation from the expected mutation frequency and number of samples per annum shows that as sample numbers increase the time taken to identify a deviation of a particular magnitude decreases, often markedly (Figure 1). Moving in increments of 200 samples, the most pronounced decrease in time is noted at the lower end of the scale i.e., increasing from 200 to 400 patients vs. increasing from 1600 to 1800 patients.

The size of the deviation too has a definite effect on the ability to detect the deviation and the time taken to do so. For example, by comparing the subplots of Figure 1, it is evident that the identification of a deviation of 10 percent would require much greater sample numbers than one would require to detect a deviation of 40 percent. This observation holds true for all prior frequency estimates tested with this model.

### SPC parameters can be used to estimate optimal/suboptimal service size

In order to forecast the required number of samples necessary to allow a laboratory to detect a deviation of a given percentage from an expected mutation frequency (also referred to as a prior frequency estimate), a quick reference chart was generated. This chart compares the expected frequency of a mutation with a percentage deviation cut-off to suggest the number of samples that might be required to detect this deviation with a power of 0.8 (Table 1). It is also possible to use this table to calculate the number of individuals likely to be unnecessarily affected by under or over treatment as a result of suboptimal service capacity. For example if Laboratory A examines 157 samples per annum it is likely to be able to detect a deviation of 30% from a prior frequency estimate of 35% i.e., an increase in mutation frequency from 35 to 45.5% of samples tested or a decrease from 35 to 24.5%. In the case of an increase from 35 to 45.5% this might potentially result in over treatment of 16 patients per annum. The laboratory would be able to detect a similar level of under treatment if the mutation detection frequency decreased. Laboratory B examines 371 samples per annum. It can detect the same 30% deviation within 5 months. Laboratory B can also detect a 20% deviation from expected values and identify a clinical risk that would not be detected by Laboratory A through annual review.

### Application of SPC to modeled data

The applicability of point frequency estimates with control data to monitoring and detection of laboratory processes is illustrated in Figure 2. The modeled data used for this chart show the type of variability one might expect from a process that is in control i.e., not subject to special cause variation. The ability of each plot (20 and 30%) to detect the commencement and continuation of a decrease from 44 to 30 percent mutation frequency is illustrated in Figure 2.

The plots also highlight the shorter time intervals and greater confidence intervals of the 30% deviation chart relative to the 20% deviation chart. From the figure it can be noted that the 30% deviation chart would be the first chart to detect the deviation from the prior frequency estimate although the 20% deviation chart may have the ability to detect deviations of lesser magnitude.

## Discussion

The utility of SPC is hard to deny, it permits a broad overview of an entire analytical process. In the context of molecular pathology and molecular oncology this means that assay deviations leading to over or under treatment of patients can be identified and any process faults may be corrected within a clinically relevant time frame. As a quality control technique SPC is in common use in many laboratories but a lack of suitable methods to apply it to qualitative data have meant that it is more often applied to quantitative methods.

Using a characteristic such as a point estimate of the mutation frequency for comparison to an expected frequency gives an estimate of the performance of a clinical laboratory testing process. However, calculations such as this are fraught with scope for mis-interpretation or inappropriate usage. For example, should one calculate such a figure with too few samples it would not be reflective of the true frequency in the population being tested. Similarly, a failure to calculate confidence intervals would give the observant little room to qualify a deviation from normal as being clinically or analytically relevant. The opposite effect might also be observed if one used too many samples for the calculation and thus delayed an opportunity to detect a process deviation.

By pre-defining an optimal sample number and prior frequency estimate for a given mutation detection assay, it is possible to use mutation frequency calculations coupled with confidence interval estimates to assess whether a process is performing as expected. The modeled data presented in Figure 2 demonstrate how a laboratory may graph mutation frequency relative to a prior frequency estimate; the estimate in this case being 44%. The modeled deviation from the “normal” mutation frequency which begins after week 52 is easily detected by the short interval of the 30% deviation chart. The 20% deviation chart by contrast takes longer to detect the deviation as the proportion of positive results across the longer time frame was not sufficient to draw the limits of normal outside of the prior frequency estimate. While this modeled data illustrates but a single example it should be robust to changes in mutation frequency and deviation size, provided sample numbers are sufficiently large. This highlights prior determination of sample size as a necessary component of this system and quick estimates of sample size can be determined using Table 1 although a more complete calculation may be required depending on circumstances.

The implications of this work are clear for laboratories processing smaller sample numbers or those laboratories testing for rare mutations. In each case the time to detect a process or assay deviation is increased, thus, in such cases measures should be taken to either increase sample throughput, or to increase the relative mutation frequency within the population being submitted for testing. It should also be of note that swelling numbers by broadening testing guidelines would have a detrimental effect on the ability of SPC to detect a deviation from a prior frequency estimate as it may decrease the relative frequency of mutations within the sample cohort. An amalgamation of test frequencies can render this approach far less informative, thus, each assay should be treated separately for the purposes of process control. Failure to do so may result in multiple deviations canceling one another out or deviations in an assay for a single marker being missed when combined with data from more numerous markers.

It must be noted that not all assays will have the ability to scale to the numbers required to implement SPC in a clinically relevant time frame. As an example, pediatric cancers may occur at an incidence of 130 to 160 cases per million children (Kaatsch, 2010). This would mean that molecular testing of childhood cancers may be too infrequent to generate a sufficient volume of data to implement this form of SPC. Thus, one should consider these guideline numbers for use when possible and realize that where assays do not fulfill sizing requirements additional EQA or confirmatory assays may be necessary to circumvent this limitation. However, guidelines for mitigating small sample numbers are absent from the literature so such mitigation would require careful implementation and possibly peer review to ensure optimal quality. If one compares pediatric cancers to colorectal cancer which have an incidence of 400 cases per million in the USA (Haggar and Boushey, 2009), and a bias toward particular predictive biomarkers such as *KRAS* or *NRAS* mutation (Douillard et al., 2013) the feasibility of applying this technique to colorectal and other common cancers is clear.

As modeled data was chosen to circumvent limitations with data availability application to historic or prospective laboratory data will be necessary to further test the method. Future work to test the utility of this assay in routine practice will be conducted by our laboratory and modification of certain parameters may be required to further optimize the system for routine use. For example, modification of the study power and chosen confidence interval may better suit some applications. Plotting of mutation frequency may lend itself to trend analysis similar to Westgard rules (Westgard et al., 1981), and depending on interpretation, a particular laboratory may favor different parameters on the X-axis e.g., sample number rather than time in weeks. The ability of SPC to act in concert with EQA schemes to provide process rather than assay control is a future consideration and any laboratory that seeks to optimize quality will require a considered approach to get an optimal return from both methodologies.

Within a modern clinical chemistry laboratory it would be surprising to find SPC absent from the daily workflow. The benefits of routine SPC are so apparent that it has seen near-universal adoption. While features such as test volume, low control to sample ratio and quantitative assays lend themselves to this process, we have established that SPC can also be applied to qualitative tests. To our knowledge this is the first report of SPC being described for use with qualitative molecular assays so it would be reasonable to expect some modifications to this system in the future which may result in improved applicability to the routine laboratory. Based on the data from our study and the obvious benefits of SPC we feel that SPC for qualitative assays should be implemented in routine practice where possible. Additionally, while our descriptions have focussed on qualitative molecular assays in cancer this approach should be broadly applicable across a range of qualitative assays.

## Author contributions

Cathal P. O'Brien: Designed the study, performed analysis, wrote and approved manuscript. Stephen P. Finn: Designed the study, wrote and approved manuscript.

### Conflict of interest statement

The authors declare that the research was conducted in the absence of any commercial or financial relationships that could be construed as a potential conflict of interest.
